# Effect of onset of type 2 diabetes on risks of cardiovascular disease and heart failure among new Zealanders with impaired glucose tolerance over 25 years: tapered-matched landmark analysis

**DOI:** 10.1186/s12933-023-01871-y

**Published:** 2023-06-30

**Authors:** Dahai Yu, Bingjie Qu, Uchechukwu Levi Osuagwu, Karen Pickering, John Baker, Richard Cutfield MBChB, Yamei Cai, Brandon J Orr-Walker, Gerhard Sundborn, Zhanzheng Zhao, David Simmons

**Affiliations:** 1 Department of Nephrology, the First Affiliated Hospital, Zhengzhou University, Zhengzhou, 450052 China; 2 Primary Care Centre Versus Arthritis, School of Medicine, Keele University, Keele, ST5 5BG UK; 3 Translational Health Research Institute (THRI), Western Sydney University, Campbelltown, Sydney, NSW 2560 Australia; 4 School of Medicine, Western Sydney University, Campbelltown, Sydney, NSW 2751 Australia; 5 Diabetes Foundation Aotearoa, Otara, New Zealand; 6 Department of Diabetes and Endocrinology, Counties Manukau Health, Auckland, New Zealand; 7 Department of Diabetes and Endocrinology, Waitemata District Health Board, Auckland, New Zealand; 8 Section of Pacific Health, the University of Auckland, Auckland, New Zealand; 9 Macarthur Clinical School, School of Medicine, Western Sydney University, Locked Bag 1797, Campbelltown, NSW 2751 Australia

**Keywords:** Impaired glucose tolerance, Type 2 diabetes, Cardiovascular diseases, Heart failure, Tapered matching, Landmark analysis

## Abstract

**Background:**

This study aimed to examine the association between the incident onset of T2DM and 5- and 10-year risks of CVD and HF in people with IGT identified in primary care in South and West Auckland, New Zealand (NZ) between 1994 and 2019.

**Methods:**

We compared CVD and HF risks in patients with IGT and with/without T2D newly diagnosed within the exposure window (1–5 years). Tapered matching and landmark analysis (to account for immortal bias) were used to control for potential effects of known confounders.

**Results:**

Among 26,794 patients enrolled with IGT, 845 had T2D newly diagnosed within 5 years from enrolment (landmark date) and 15,452 did not have T2D diagnosed. Patients progressing to T2D (vs. those not progressing) had a similar 5-year risk for CVD (hazard ratio 1.19; 95% CI 0.61–2.32) but significantly higher 10-year risk of CVD (2.45(1.40–4.29)), 5-year risk of HF (1.94(1.20–3.12)) and 10-year risk of HF (2.84(1.83–4.39). The association between the onset of T2D and risk of 10-year risk of CVD, 5-year and 10-year risk of HF was more likely among men, the socioeconomically deprived, those currently smoking, patients with higher metabolic measures and/or those with lower renal function. Patients of NZ European ethnicity had a lower 10-year risk of CVD.

**Conclusions:**

The study suggests that the diagnosis of T2D mediates the risk of CVD and HF in people with IGT. The development of risk scores to identify and better manage individuals with IGT at high risk of T2D is warranted.

**Supplementary Information:**

The online version contains supplementary material available at 10.1186/s12933-023-01871-y.

## Background

Globally, over recent decades in the general population, glycaemia has rapidly increased across high, medium and low-income countries [1]. A meta-analysis from 2011 found a 0.1 mmol/L increase in fasting plasma glucose (FPG) compared to 1980, with an average FPG of 5.4 mmol/L for women and 5.5 mmol/L for men. Individuals living in Oceania had the highest average FPG level at 6.1 mmol/L both for women and men [1].

The growth in glycaemia has resulted in a rise in the prevalence of prediabetes including impaired glucose tolerance (IGT) [2]. The National Health and Nutrition Examination Survey (NHANES) found that 35% of adults aged 20 years and 50% of those over 65 had prediabetes between 2005 and 2008 [3]. In New Zealand (NZ), a 2008–2009 national survey estimated the prevalence of prediabetes at 25.5% among those aged 15 years and over [4].

Based on previous studies suggesting that the mild hyperglycaemia of IGT is sufficient to affect the vasculature and thereby lead to their excess prevalence of coronary disease [5, 6], several studies have explored the incidence of newly diagnosed type 2 diabetes (T2D) in individuals with prediabetes (IGT and IFG) [7–9], as well as the associated risks of cardiovascular disease (CVD) and heart failure (HF) in this population [10–13]. For example, in United States population-based cohorts, prediabetes (defined as an FPG concentration of 5.6–6.9 mmol/L) was associated with an increased lifetime risk of heart failure (HF) in middle-aged White men and women and Black women [13]. A meta-analysis revealed that individuals with prediabetes had a higher risk of HF compared to those with normoglycemia [10].

However, there is a scarcity of studies investigating the impact of T2D onset on future CVD and HF risk in individuals with IGT [14] [15], and It is also unknown whether among individuals with IGT, the risk of future CVD differs between those with and without incident T2D, if their conventional known CVD risk factors (i.e., demographic factors, lifestyle risk factors, metabolic measures, and treatments) are comparable.

To explore these questions, we undertook this study to compare the 5- and 10-year risk of incident CVD and HF between individuals with and without onset of T2D in the IGT population, one subtype of prediabetes, with matching conventional CVD risk factors using a matched cohort derived from longitudinal prediabetes audit data linked with national registration databases in NZ.

## Methods

### Data source

The Diabetes Care Support Service (DCSS) was established in 1991 with the aim of improving diabetes care standards in South, East and West Auckland through general practice audits [16]. The de-identified DCSS database was linked with various data sources, including national cancer and death registration, hospitalisation, pharmaceutical claim, and socioeconomic status (SES). This allowed us to identify a cohort of patients aged ≥ 18 years with IGT in Auckland, NZ.

The IGT was diagnosed based on the 2-hour glucose of 7.8–11 mmol/L on an oral glucose tolerance test (OGTT) [17]. The final cohort data included demographics, diabetes clinical data (including smoking, body mass index [BMI], blood pressure [BP], HbA1c, lipids) and clinical treatment (antihypertensive, statin, antiplatelet and/or anticoagulant treatment). These data have been validated through enumeration assessment and internal quality control policies with auditors regularly cross checking, random and routine sampling/checking of data entry, and active data management (e.g., queries, checking unusual numbers, ranking of columns, duplicate checking)[16, 18, 19]. Pharmaceutical claims data includes all prescriptions issued for patients and was used to cross-validate the prescription data in DCSS. Only pharmaceutical claims data after 2006 were available for data linkage as National Health Index numbers were not universal before 2006. Data for all patients from their first DCSS enrolment date were included with the last enrolment on 31/7/2018.

The North Health Ethics Committee approved the DCSS for research purposes in 1992, and then as an ongoing audit in 1996 (92/006). Ethics review was waived by the New Zealand Health and Disability Ethics Committees on March 25, 2019. Anonymised data were used for this analysis. Signed consent to participate was provided by an authorised signatory for each general practice. This manuscript reporting study findings were written in adherence with the Strengthening the Reporting of Observational Studies in Epidemiology (STROBE) reporting guideline.

### Exposure

We classified patients with IGT by exposure to T2D. Newly diagnosed T2D was defined as the earliest diagnosis of T2D identified within the nationally linked datasets during the exposure window. This includes diagnoses based on the primary International Classification of Diseases, Ninth Revision (ICD-9) and ICD-10 codes recorded in outpatient and inpatient databases, primary care records, or the recording of anti-diabetes prescriptions recorded in the pharmaceutical database.

In this study, we utilised a landmark analysis to examine the effect of the onset of T2D on risk of CVD or HF. In this landmark analysis, a fixed time after cohort entry was selected a priori to conduct a survival analysis [20]. Only patients with IGT alive at the landmark date were included, and the onset of T2D was based on exposure before the landmark date. Exposure was only evaluated between the index date and the landmark time point (exposure window), and the outcome was then assessed from the landmark time point [21]. Five landmark time points were determined a priori in this study, specifically at 1, 2 ,3, 4, and 5 years after the cohort enrolment date. Exposure status was assigned for patients with IGT who were still alive at the landmark dates.

### Outcomes

Outcomes included an incident CVD and HF event. Both CVD and HF events were defined as incident clinical events recorded in the linked national outpatient, inpatient, and death registration databases by the primary ICD-9 and ICD-10 codes (Supplemental Table 1) during the follow-up period since the landmark time point. Patients with IGT were followed up from the landmark date until an outcome of interest occurred, or until December 31, 2019 for those without any outcome of interest.

### Covariates

Covariates included patient characteristics, lifestyle factors, clinical measurements, antihypertensive, anticoagulant, and lipid-lowering treatments at enrolment. The area deprivation indicator, the NZDep2013 Index of Deprivation, was used to define socioeconomic status [22]. The NZDep2013 provides an Index of Multiple Deprivation (IMD) score for each New Zealand meshblock (geographical unit containing a median of 81 people) [22]. Scores on the NZDep2013 scale of deprivation range from 1 to 10, with lower scores indicating less deprivation; the scale divides New Zealand into tenths of the distribution of the first principal component scores and was consistent with prior deprivation measures [23]. To maintain statistical power, the IMD was redefined by recategorizing the NZDep2013 into 5 groups: IMD-1 (least deprived: NZDep2013 scores of 1–2); IMD-2, IMD-3, and IMD-4 (scores of 3–4, 5–6, and 7–8, respectively); and IMD-5 (most deprived: scores of 9–10).

### Statistical analysis

We utilised tapered matching methods to account for confounding [24]. This method assessed the effect of the onset of T2D on CVD or HF risks between the focal (exposed: IGT with the onset of T2D during the exposure time window) and control (IGT without the onset of T2D during the exposure time window) groups via entropy balancing which involved incrementally matching the control cohort to the focal cohorts using additional covariates and directly observing how the matched cohort changed in terms of hazard ratios (HRs) and in terms of unmatched covariates.

Before tapered matching and balancing, to minimise model dependence and the potential for irresolvable imbalances between the comparative groups, we used coarsened exact matching (CEM) to restrict the comparison of patients in comparative groups to areas of common support [25]. During each landmark analysis (from 1-year landmark analysis to 5-year landmark analysis), a series of 10 matching steps were conducted, with patients with IGT in the comparative groups being retained after the completion of the 10th matching step. (Supplemental Figs. 1–5).

After excluding participants with no areas of common support, we used entropy balancing to minimize differences in the distribution of matching variables between comparison groups. Entropy balancing involves maximum entropy reweighting of the unexposed group (in the present study, the group without the onset of T2D) by directly incorporating covariable balance into the weight function, in which the matched sample is reweighted in each matching step to key target moments (mean, variance, and skewness) [26]. All pre-processing (both CEM and entropy balancing) was performed without reference to outcomes.

Weighted Cox proportional hazards regression incorporating matching weights estimated from each matching step by entropy matching, and accounting for competing risk of all death (except deaths due to the incident CVD/HF events), was applied in each matching step to provide an estimate of the relative risk of outcomes between comparison groups. The risks of incident outcomes between comparative groups were estimated from the same model. Outcome of CVD and HF was evaluated separately. Data on each variable were missing in fewer than 6% of eligible cohort members. Based on the worst scenario of 6% of patients with 1 more missing data, 6 imputed dataset were created for multiple imputation with chained equation, and estimations were made by Robin’s rule [27]. Analyses were conducted using Stata/MP, version 17.0 (StataCorp LLC). Statistical significance was set at 2-tailed P < 0.05.

## Results

Overall, 26,794 patients with IGT were enrolled by the DCSS between 1994 and 2018.

Patients with death or loss of follow-up or occurrent of outcomes between the enrolment date and the landmark time point were excluded. After, 10 matching steps were processed to create matched cohorts of patients with and without the onset of T2D (Supplemental Figs. 1–5): 157 cases vs. 2,019 controls for 1-year, 332 vs. 3,931 for 2-year,508 vs. 5,163 for 3-year, 636 vs. 5,341 for 4-year, and 697 vs. 4,603 controls for 5-year landmark analysis.

Characteristics of people with IGT with and without the onset of T2D before and after matching are shown in Supplemental Tables 1 and Table 2. For 1-, 2-, 3-, 4-, and 5-year landmark analysis. After tapered matching (especially entropy matching), no significant differences were identified in variables incorporated in the matching process between people with IGT with and without the onset of T2D, confirming the success of our matching process (Table 1).

**Table 1:** Comparison of patients with and without the onset of type 2 diabetes among patients with impaired glucose tolerance in the final entropy balancing matched cohorts. *N stands for the sample size for the cohorts before matching; n stands for the sample size for the cohort after matching. Continuous variables were presented as weighted means (standard error). Categorical variables were presented as weighted prevalence (standard error). IMD indicates the index of multiple deprivation*

	1-year landmark			2-year landmark			3-year landmark			4-year landmark			5-year landmark		
	Without T2D onset	With T2D onset	*P*-value	Without T2D onset	With T2D onset	*P*-value	Without T2D onset	With T2D onset	*P*-value	Without T2D onset	With T2D onset	*P*-value	Without T2D onset	With T2D onset	*P*-value
No of the unmatched cohort	N = 24,893	N = 180		N = 24,575	N = 381		N = 22,703	N = 589		N = 19,597	N = 749		N = 15,452	N = 845	
No of the final matched cohort	n = 2019	N = 157		n = 3931	n = 332		n = 5163	n = 508		n = 5341	n = 636		n = 4603	n = 697	
Age, years	57.2 (13.3)	58.2 (12.6)	0.506	57.1 (12.5)	58.6 (11.5)	0.114	56.7 (12.4)	58.1 (12.4)	0.087	56.3 (12.3)	57.5 (12.1)	0.076	56.5 (12.0)	57.2 (12.3)	0.342
Female Gender	62.5 (0.03)	62.7 (0.05)	0.965	54.5 (0.02)	54.5 (0.03)	0.975	51.7 (0.02)	51.7 (0.02)		52.9 (0.01)	52.9 (0.03)	0.981	52.6 (0.01)	52.6 (0.01)	0.983
New Zealand European	47.8 (0.03)	47.8 (0.05)	0.990	45.9 (0.02)	45.9 (0.02)	0.993	44.2 (0.02)	44.2 (0.03)	0.994	44.1 (0.01)	44.1 (0.03)	0.995	44.1 (0.01)	44.1 (0.02)	0.995
Enrol cohort															
1994–1998	1.2 (0.01)	2.1 (0.01)	0.578	0.6 (0.004)	1.0 (0.007)	0.813	0.6 (0.003)	0.6 (0.004)	0.999	0.8 (0.006)	0.5 (0.004)	0.871	0.8 (0.006)	0.5 (0.003)	0.682
1999–2003	8.2 (0.02)	4.3 (0.02)		7.9 (0.02)	6.1 (0.02)		6.9 (0.01)	6.8 (0.01)		6.2 (0.01)	7.3 (0.01)		6.5 (0.01)	8.0 (0.01)	
2004–2008	15.0 (0.02)	20.2 (0.04)		16.0 (0.02)	18.4 (0.03)		15.5 (0.01)	15.5 (0.02)		15.8 (0.01)	14.0 (0.02)		18.5 (0.01)	16.1 (0.02)	
2009–2013	46.9 (0.03)	43.6 (0.05)		45.4 (0.02)	43.9 (0.04)		46.6 (0.01)	46.6 (0.03)		52.3 (0.01)	53.5 (0.03)		60.9 (0.02)	62.5 (0.02)	
2014–2018	28.8 (0.02)	29.8 (0.05)		30.2 (0.01)	30.6 (0.03)		30.3 (0.01)	30.4 (0.03)		24.9 (0.01)	24.7 (0.02)		13.2 (0.01)	12.9 (0.02)	
IMD group (NZDep13 scale)															
Least deprivation: IMD-1 (1 or 2)	7.1 (0.01)	7.4 (0.03)	0.983	7.4 (0.01)	7.1 (0.02)	0.978	8.7 (0.01)	8.8 (0.02)	0.998	9.6 (0.01)	9.9 (0.02)	0.904	9.0 (0.007)	9.0 (0.01)	0.999
IMD-2 (3 or 4)	13.4 (0.02)	11.7 (0.03)		16.0 (0.01)	16.8 (0.03)		16.4 (0.01)	15.9 (0.02)		15.9 (0.01)	14.8 (0.02)		15.9 (0.01)	15.9 (0.01)	
IMD-3 (5 or 6)	12.9 (0.03)	14.8 (0.04)		14.7 (0.02)	13.3 (0.02)		11.2 (0.01)	11.8 (0.02)		10.9 (0.01)	12.5 (0.02)		11.5 (0.01)	11.5 (0.01)	
IMD-4 (7 or 8)	20.0 (0.02)	19.1 (0.04)		15.1 (0.01)	16.3 (0.03)		15.4 (0.01)	15.2 (0.02)		15.5 (0.01)	14.5 (0.02)		15.7 (0.01)	15.8 (0.01)	
Most deprivation: IMD-5 (9 or 10)	46.6 (0.03)	46.8 (0.05)		46.7 (0.02)	46.4 (0.04)		48.3 (0.01)	48.3 (0.03)		48.1 (0.01)	48.3 (0.03)		47.8 (0.01)	47.8 (0.02)	
Smoking status															
Never smoking	55.6 (0.03)	55.3 (0.05)	0.994	51.2 (0.02)	51.0 (0.04)	0.998	53.4 (0.02)	53.4 (0.03)	0.999	53.1 (0.01)	53.0 (0.03)	0.999	52.9 (0.01)	52.9 (0.02)	0.999
Ex-smoker	27.1 (0.03)	27.7 (0.05)		33.4 (0.02)	33.7 (0.03)		30.9 (0.01)	31.0 (0.03)		30.3 (0.01)	30.4 (0.02)		30.2 (0.01)	30.3 (0.02)	
Current Smoker	17.2 (0.02)	17.0 (0.04)		15.4 (0.01)	15.3 (0.03)		15.6 (0.01)	15.5 (0.02)		16.7 (0.01)	16.6 (0.02)		16.8 (0.01)	16.8 (0.02)	
Body mass index, kg/m^2^	33.0 (7.1)	33.1 (7.1)	0.951	33.2 (6.9)	33.2 (6.9)	0.962	33.4 (7.0)	33.4 (7.0)	0.967	33.3 (6.7)	33.3 (6.7)	0.970	33.7 (6.7)	33.7 (6.7)	0.972
Systolic blood pressure, mmHg	133 (18)	133(16)	0.166	133 (17)	133 (17)	0.437	133 (17)	133 (17)	0.433	133 (17)	133 (17)	0.315	133 (17)	133 (17)	0.245
Diastolic blood pressure, mmHg	80 (11)	80 (10)	0.180	80 (10)	80 (10)	0.670	80 (10)	80 (10)	0.702	80 (10)	80 (10)	0.737	81 (10)	81 (10)	0.618
HbA1c, mmol/mol	42.9 (4.0)	42.9 (4.0)	0.897	43.5 (3.9)	43.5 (3.9)	0.918	44.2 (4.2)	44.2 (4.2)	0.934	44.4 (4.0)	44.4 (4.0)	0.939	44.7 (3.8)	44.7 (3.8)	0.940
Total cholesterol, mmol/L	4.8 (0.8)	4.8 (0.8)	0.941	4.8 (0.9)	4.8 (0.9)	0.959	4.8 (0.9)	4.8 (0.9)	0.964	4.8 (0.9)	4.8 (0.9)	0.968	4.8 (0.9)	4.8 (0.9)	0.971
Triglyceride, mmol/L	1.6 (0.8)	1.6 (0.6)	0.398	1.8 (0.8)	1.8 (0.8)	0.506	1.7 (0.8)	1.7 (0.8)	0.674	1.8 (0.8)	1.8 (0.8)	0.571	1.7 (0.8)	1.7 (0.8)	0.735
Low-density lipoprotein cholesterol, mmol/L	2.7 (0.7)	2.7 (0.7)	0.961	2.6 (0.7)	2.6 (0.7)	0.973	2.7 (0.7)	2.7 (0.7)	0.975	2.7 (0.8)	2.7 (0.8)	0.979	2.7 (0.8)	2.7 (0.8)	0.980
High-density lipoprotein cholesterol, mmol/L	1.3 (0.4)	1.3 (0.4)	0.746	1.3 (0.4)	1.3 (0.4)	0.747	1.3 (0.4)	1.3 (0.4)	0.951	1.2 (0.4)	1.2 (0.4)	0.971	1.2 (0.4)	1.2 (0.4)	0.363
estimated Glomerular filtration rate < 90 ml/min/1.73 m^2^	36.5 (0.02)	36.2 (0.05)	0.954	33.5 (0.02)	33.7 (0.03)	0.998	34.3 (0.01)	34.4 (0.03)	0.999	38.6 (0.01)	38.7 (0.02)	0.999	59.2 (0.01)	59.3 (0.02)	0.999
Antihypertensive treatment	29.7 (0.03)	29.7 (0.05)	0.994	29.1 (0.02)	29.1 (0.02)	0.995	31.4 (0.02)	31.4 (0.03)	0.996	31.4 (0.02)	31.4 (0.02)	0.996	36.8 (0.02)	36.8 (0.02)	0.996
Statin treatment	28.7 (0.03)	28.7 (0.05)	0.994	27.5 (0.02)	27.5 (0.02)	0.995	28.4 (0.02)	28.4 (0.03)	0.996	29.3 (0.02)	29.3 (0.02)	0.996	34.2 (0.02)	34.2 (0.02)	0.996
Antiplatelet or anticoagulant treatment	3.2 (0.02)	3.2 (0.02)	0.998	15.3 (0.01)	15.3 (0.01)	0.999	1.0 (0.004)	1.0 (0.006)	0.999	0.8 (0.033)	0.8 (0.004)	0.999	0.9 (0.003)	0.9 (0.004)	0.999

As presented in Tables 2 and 5-year risk decreased across the 1–4 year landmark analyses (44.44 (95% confidence interval (CI): 21.31–81.73) to 18.09 (10.72–28.59) per 1,000 person-years for CVD; 19.14 (9.89–33.43) to 13.46 (7.17–23.01) per 1,000 person-years for HF) and increased in the 5-year landmark analysis (28.37 (18.18–42.21) and 17.99 (10.07–29.66) per 1,000 person-years for CVD and HF, respectively) in the focal population, whereas the 5-year risk increased from 1 to 5 year landmark analysis both for CVD and HF (3.46 (1.73–6.19) to 9.46 (7.37–11.95) per 1,000 person-years for CVD; 4.17 (2.89–5.83) to 6.03 (4.38–8.09) per 1,000 person-years for HF) in the matched control population.

**Table 2:** 5-year and 10-year rates of cardiovascular disease among cases compared between people with IGT with and without the onset of type 2 diabetes after coarsened and exact matching for 1–5 year landmark analysis

	5-year risk		10-year risk	
	Cases: with onset of T2D	Controls: without onset of T2D	Cases: with onset of T2D	Controls: without onset of T2D
	Rate (95% CI), per 1,000 person-years	Rate (95% CI), per 1,000 person-years	Rate (95% CI), per 1,000 person-years	Rate (95% CI), per 1,000 person-years
	**Cardiovascular Diseases**			
**1-year landmark analysis**	44.44 (21.31 to 81.73)	3.46 (1.73 to 6.19)	23.13 (12.32 to 39.56)	3.50 (2.38 to 4.97)
**2-year landmark analysis**	20.73 (11.04 to 35.46)	3.80 (2.58 to 5.40)	22.50 (14.27 to 33.77)	4.49 (3.44 to 5.75)
**3-year landmark analysis**	19.86 (11.57 to 31.80)	4.30 (3.15 to 5.74)	27.42 (18.99 to 38.32)	5.74 (4.57 to 7.12)
**4-year landmark analysis**	18.09 (10.72 to 28.59)	5.42 (4.08 to 7.05)	26.90 (18.63 to 37.59)	6.98 (5.62 to 8.58)
**5-year landmark analysis**	28.37 (18.18 to 42.21)	9.46 (7.37 to 11.95)	36.87 (26.58 to 49.84)	9.34 (7.52 to 11.47)
	**Heart Failure**			
**1-year landmark analysis**	19.14 (9.89 to 33.43)	4.17 (2.89 to 5.83)	13.70 (7.49 to 22.98)	2.97 (2.13 to 4.03)
**2-year landmark analysis**	18.27 (9.12 to 32.69)	8.27 (6.41 to 10.50)	14.42 (7.88 to 24.19)	5.31 (4.17 to 6.67)
**3-year landmark analysis**	12.03 (5.77 to 22.13)	5.76 (4.40 to 7.40)	12.69 (7.25 to 20.61)	4.75 (3.70 to 6.00)
**4-year landmark analysis**	13.46 (7.17 to 23.01)	5.18 (3.87 to 6.80)	14.86 (9.32 to 22.51)	4.67 (3.59 to 5.98)
**5-year landmark analysis**	17.99 (10.07 to 29.66)	6.03 (4.38 to 8.09)	18.44 (12.35 to 26.48)	4.88 (3.63 to 6.42)

The 10-year risk of CVD and HF increased across the 1–5 year of landmark analyses both for focal and control populations: the 10-year risk of CVD increased from 23.13 (12.32–39.56) to 36.87 (26.58–49.84) person-years in the focal population and 3.50 (2.38–4.97) to 9.34 (7.52–11.47) per 1,000 person-years in the control population; 10-year risk of HF increased from 13.70 (7.40-22.98) to 18.44 (12.35–26.48) per 1,000 person-years in the focal population and 2.97 (2.13–4.03) to 4.83 (3.63–6.42) per 1,000 person-years in the control population.

**Fig. 1:**
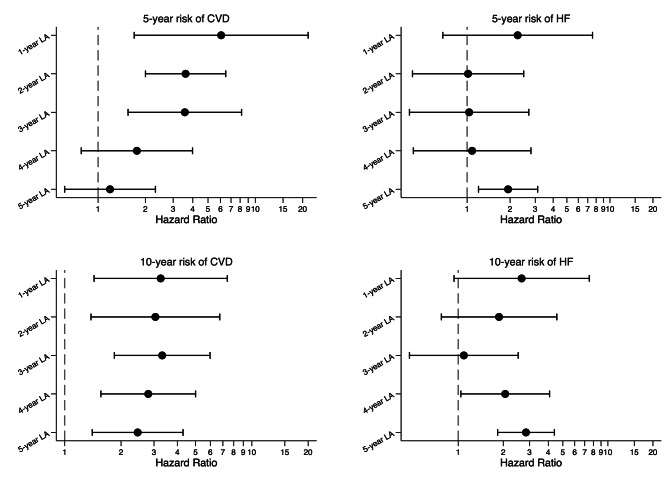
Adjusted hazard ratio for 5- and 10-year risk of cardiovascular diseases and heart failure at 1-, 2-, 3-, 4-, and 5-year landmark (final tapered matched models)

The final (by step-10 matching) adjusted hazard ratios (HRs) of 5-year risk of CVD comparing those people with and without the onset of T2D decreased from 6.06 (1.70-21.67) for the 1-year to 1.19 (0.61–2.32) for the 5-year landmark analysis (Figure 1; Supplemental Fig. 6). The final adjusted HRs of the 10-year risk of CVD comparing those people with and without the onset of T2D remained stable from 3.26 (1.44–7.39) for the 1-year to 2.45 (1.40–4.29) the 5-year landmark analysis (Figure 1; Supplemental Fig. 7).

The final adjusted HRs of 5-year risk of HF comparing those people with and without the onset of T2D decreased slightly from 2.26 (0.68–7.55) for the 1-year to 1.94 (1.20–3.12) for the 5-year landmark analysis (Figure 1; Supplemental Fig. 8). The final adjusted HRs of 10-year risk of HF comparing those people with and without the onset of T2D varied from 2.65 (0.94–7.51) for the 1-year to 2.84 (1.83–4.39) for the 5-year landmark analysis (Figure 1; Supplemental Fig. 9).

**Fig. 2:**
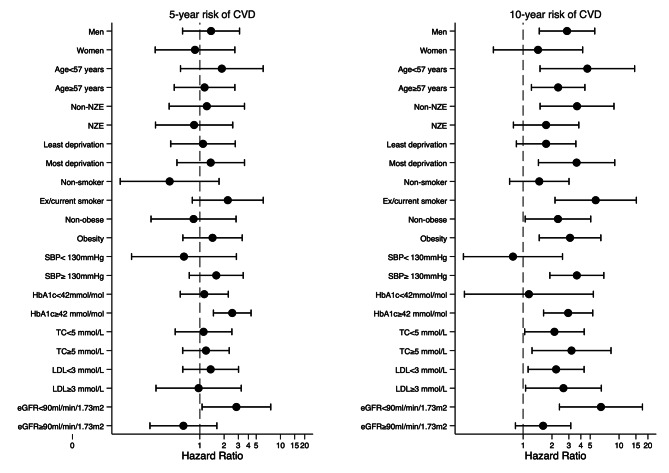
Stratified adjusted hazard ratio for 5- and 10-year risk of cardiovascular diseases at 5-year landmark (final tapered matched models)

**Fig. 3:**
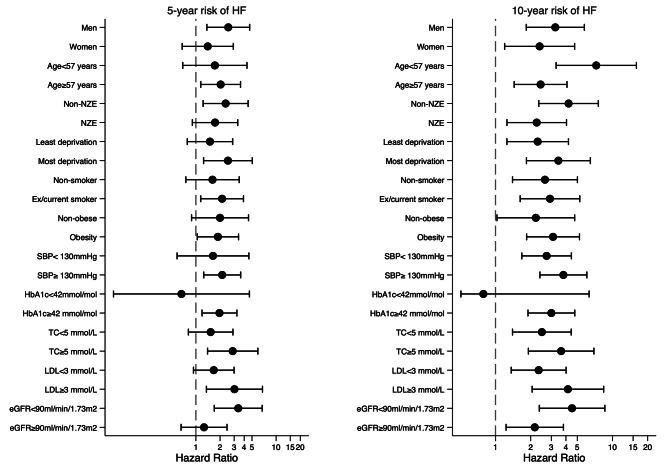
Stratified adjusted hazard ratio for 5- and 10-year risk of heart failure at 5-year landmark (final tapered matched models)

In a stratified 5-year analysis, the risk of CVD for patients with IGT with and without onset of T2D was not significantly different by sex, age, ethnicity, deprivation, smoking, obesity, SBP, TC, and LDL, but higher for those with HbA1c ≥ 42mmol/mol/6.0% and eGFR < 90ml/min/1.73 m² (Figure 2). The HR for 5-year HF risk was significantly higher in men, older people, non-NZE, the most deprived, smokers, obese people, and those with higher SBP, HbA1c, TC, LDL, and lower eGFR (Figure 3). The adjusted HR for 10-year CVD risk was significant for men, non-NZE, most deprived, smokers, both obese and non-obese, higher SBP and HbA1c, and lower eGFR. For patients with high or low lipids (TC, LDL), adjusted HRs for 10-year CVD risk were significant (Figure 2). The adjusted HR for 10-year HF risk was significant for all groups except HbA1c < 42mmol/mol/6.0% (Figure 3).

## Discussion

This population-based cohort derived from the multiple linked national databases of patients with IGT revealed that the onset of T2D within 1 to 5 years was associated with an increased 5-year and 10-year risk of HF and increased 10-year risk of CVD in patients who survived to the date of the landmark time point. The most affected were male and those who were socioeconomic deprived, smoking, obese, with higher blood pressure, HbA1c, lipids and/or reduced renal function. Given the many well-established health benefits of improving modifiable risk factors of T2D, the prevention of T2D in patients with IGT is likely to prevent CVD at the population-level. An OGTT should be considered in the T2D prevention program for individuals who are willing to undergo it, as relying solely on HbA1c as the screening strategy in the T2D prevention program may exclude many individuals with IGT. Other CVD prevention strategies beyond lifestyle should be applied to those with IGT at high-risk of developing T2D.

This study used a well-defined cohort of people with IGT to quantify the influence of incident T2D developing on the CVD and HF risk. Although all patients with IGT have been examined to have T2D or not by the index date, using the index date to start the follow-up for a later diagnosis of T2D is prone to immortal time bias, which could confer a spurious survival bias to the T2D onset group [28]. A landmark analysis, as used in the current study, has been devised to avoid this [20]. Another important factor that could influence the comparison of survival function between patients exposed and those not exposed to the onset of T2D is confounding by indication, which results from the non-random likelihood of the onset of T2D. The onset of T2D is more likely to happen among people with IGT of more advanced age, comorbidities and polypharmacy. To minimise confounding bias, we used the tapered matching method to balance the probability of the onset of T2D within the 1- to 5-year exposure time window. Both native unadjusted analysis and the tapered matching method yielded a consistent influence of the onset of T2D on long-term risk of CVD. Hypertension, dyslipidemia, and body weight are important risk factors that potentially impact the association between the onset of T2D and the risk of CVD and HF. Although these risk factors were not directly balanced during the matching process for confounder control, surrogate variables including blood pressure, antihypertensive prescriptions, lipid profiles, statin prescriptions, and body mass index were balanced in the final matched comparative cohorts.

A recent meta-analysis concluded that among individuals without diabetes, those in the highest vs. the lowest category of blood glucose level had a relative risk of CVD of 1.26 (95% CI:1.11–1.43) [29]. However, few studies have compared the risk of future CVD between those with and without incident T2D among people with prediabetes including IGT. Our study revealed that those with existing IGT who later developed T2D (vs. those who did not) within 5-years had a similar 5-year risk of CVD but a 2.5-fold 10-year risk of CVD.

A previous Swedish cohort showed that among participants with normoglycemia, the risk of HF ranged from 4.7 (American Diabetes Association (ADA) definition) to 5.0 (World Health Organisation (WHO) definition) per 1,000 person-years over 9 years follow-up, whereas participants with prediabetes (either with or without the onset of T2D), the risk of HF ranged from 6.2(5.5-7.0) (ADA definition) to 7.3(6.2–8.6) (WHO definition) per 1,000 person-years [30]. In our study, for people with IGT without the onset of T2D by the 5-year landmark time, the future 10-year risk of HF was 4.9 per 1,000 person-years: close to the estimations in the Swedish normoglycemia population. Conversely, people with IGT developing T2D within 5 years had a risk of HF of 18.4 per 1,000 person-years: higher than the Swedish cohort with pre-diabetes where there was no separation into those with or without onset the development of T2D. Our data suggest that the risk of HF is similar between those with normoglycemia and IGT without the subsequent development of T2D: the risk of HF might only increase once IGT progress to T2D.

The incidence rate of progression to T2D in the current study was 13% by the 5-year landmark time: higher than the 8% found in the ARIC cohort [31]. The ARIC cohort was older (mean age 75 years) and had a higher white ethnicity (83% of white ethnicity) than the current cohort (mean age 56 years and 44% of NZE). A systematic review incorporating adults aged 30–77 years across multiple -ethnicities found that the 5-year cumulative incidence of T2D ranged between 5 and 45% among those with IFG and ranged between 7 and 89% for those with IGT [32]. This variation in the risk of onset of T2D by age and ethnicity might indicate a differential risk of CVD and HF in the population with prediabetes [32].

In the current study, the onset of T2D was associated with a higher 10-year risk of CVD in younger, male, socioeconomically deprived individuals, as well as those with unhealthy lifestyles and metabolic issues. However, the association between the onset of T2D and the 10-year risk of HF was consistent across age, sex, socioeconomic status, lifestyle status, and clinical measurements, suggesting that the onset of T2D could drive an increased risk of HF in patients with IGT compared to those with normoglycemia [13].

Our findings highlight the importance from a CVD/HF prevention perspective, of intensive lifestyle modification and/or pharmacotherapy to reduce the incidence of T2D in the prediabetes population. There is clear evidence that intensive lifestyle modification (diet, exercise, weight loss) can prevent progression from IGT to T2D, as seen in the Swedish Malmo study (50%) [33], Finnish Diabetes Prevention Study (58%)[34] and the US Diabetes Prevention Program study (58%) [35, 36]. This protection was maintained over the long-term [37]. Conversely, metformin was less effective than lifestyle change in improving CVD risk factors[38] and has been deemed to be less cost-effective [39]. Some new drugs like glucagon-like peptide 1 (GLP-1) agonists also reduce cardiometabolic risk [40], but their role has not yet been defined beyond supporting weight reduction [41]. The early identification and comprehensive management of IGT, including adherence to lifestyle modification and appropriate medical interventions (e.g. metformin therapy), could have influenced the risk of cardiovascular events and heart failure in the population with IGT. However, local guidelines did not recommend the use of metformin with IGT and this only occurred rarely (< 1%). These interventions may have contributed to reducing the risk of progression from IGT to T2D and subsequently mitigated the associated cardiovascular risks. Investigation into the impact of specific treatment modalities on cardiovascular outcomes is warranted in future studies.

Immortal bias is more difficult to identify than confounding effects by indication. For example, the onset of T2D was associated with a differential effect on CVD and HF risk in those with IGT when comparing survival in patients with and without the onset of T2D. Using the T2D onset time to replace the original index date, means the focal population survived from the original index date to the T2D onset date, whereas the matched control population could still have been at an early stage of the original index date when applying the onset time as the index date to follow up. Although the index date was matched between patients with and without incident T2D, it did not mean that the time from the index date to the date of onset of T2D was matched between the two groups. The patients with T2D developing were still more likely to have a spurious survival advantage (to be alive by the diagnosis of CVD or HF in the future) because by design they had to survive to the date of onset of their T2D in order to be assigned as cases. Therefore, without tackling immortal bias by design (such as using a landmark analysis), a biased estimate would inevitably occur.

The matching process within the 1–5 year landmark period allows patients in the group without the onset of T2D have the possibility of developing to T2D beyond 5 years. We did not apply a longer landmark period to avoid potential survival bias introduced by excluding patients with outcomes within the extended landmark period. This approach ensures that the comparable samples better represent the IGT populations in the real-world setting and maintains the integrity of the association between the onset of T2D and outcomes. Matching follow-up time between enrolment and the onset of T2D could be considered as an optional method to mitigate immortal bias. However, implementing this method may pose challenges to further tapered matching, which aims to establish comparability between cohorts on each variable. Future studies with larger sample sizes could explore the incorporation of this method.

Our study has several strengths, including being the largest study of a multi-ethnic matched cohorts of participants with IGT in NZ and one of the largest IGT cohorts globally to investigate the association between the onset of T2D and 5- and 10-year risk of CVD and HF. These cohorts included all patients from participating general practices and used large, nationally representative databases to follow patients prospectively and track all incident CVD and HF. All CVD and HF used in this study were based on the linkage of hospitalisation and death registration datasets, which had good validation of outcomes. The exposure and outcome definitions relied on electronic health records from the national linked primary care and secondary care databases, registry dataset, and pharmaceutical database. Considering the country’s advanced position in processing electronic healthcare data within the national healthcare system, the validity of the individual electronic healthcare data has been identified through national audits, and its precision is further enhanced when using linked datasets. Moreover, strict internal data validation processes were applied in this study to ensure the high validity of the exposure and outcomes [42]. Second, the use of landmark analysis within 1- to 5-year time windows provided a robust methodology to rule out immortal bias. Another key strength of this work in methodology was the application of an innovative, tapered matching method to form ‘quasi-trial’ comparison cohorts between patients with IGT with and without an onset of T2D to compare the risk of 5- and 10-year risk of CVD and HF and transparently examine how differences in specific sets of confounders contributed to the risks of CVD and HF. The study limitations include the national representativeness of the sample and of the participating general practices in NZ. Having impaired fasting glucose (IFG) among patients could potentially introduce bias in the estimations. Although the prevalence of IFG was not considered in the matching process, the prevalence of IFG between the comparative samples was comparable in each landmark analysis. There may be potential differential risks between detailed subgroups within the non-NZE grouping. However, previous studies have suggested that ethnic disparities in cardiovascular events are significantly driven by socioeconomic status, which is associated with lifestyle risk factors [43, 44]. In our study, we incorporated 5-level socioeconomic status and lifestyle risk factors such as smoking status in the matching process. We also conducted subgroup analyses to observe the risk differentiation based on socioeconomic status and smoking status. Moreover, including multiple ethnicity categories would complicate the matching process, resulting in unmatched or very small comparative cohorts with lower statistical power and higher uncertainty in the estimates. Therefore, the current sample was unable to investigate the multi-ethnic level association between the onset of T2D and the risk of outcomes. Future studies with larger sample sizes, particularly encompassing a diverse range of ethnicities, are warranted to estimate ethnic-specific associations, enhance our understanding of ethnic differences in risk, and facilitate the development and implementation of targeted ethnic-specific interventions. Information on certain risk factors for CVD and HF examined in this study was not available, including dietary information, physical activity and genetic variants, which could have influenced the association between T2D onset and the outcomes. Future studies should take these risk factors into account. Although the national linked data used in the current study exhibit high precision, further validation of the associations should be pursued through external studies using electronic health record data from other countries. Our study focused on the association between impaired glucose tolerance progressing to type 2 diabetes and subsequent CVD and heart failure. We recognize that comprehensive management of multiple risk factors, such as adopting a healthy diet, engaging in regular physical activity, weight management, making lifestyle changes like smoking cessation and limiting alcohol consumption for CVD prevention [45], *and early identification and management of risk factors such as hypertension, dyslipidemia, and obesity to prevent the progression to heart failure* [46], *should be considered as effective prevention strategies by health policymakers. Future research and interventions (e.g. benefits from GLP1 Receptor agonists) should aim to evaluate the implementation and effectiveness of these recommendations in reducing the burden of CVD and heart failure in the NZ population with IFG.*

## Conclusions

In conclusion, this tapered-matched landmark analysis in a large cohort with IGT found an increased 5-year risk of HF, and 10-year risks of CVD and HF from the onset of T2D at 5 years after enrolment. The risk of CVD and HF among those with IGT without the onset of T2D was comparable to previous estimations in a normoglycaemia population. The association between the onset of T2D and the risk of CVD is more certain in those with existing risk factors. The association between the onset of T2D and HF risk is very consistent over different risk factors. The increased risk of CVD and HF in IGT might be mediated by the onset of T2D. Future intervention studies of applying evidence-based lifestyle interventions and pharmaceutical interventions (e.g. GLP-1) in those with IGT as early as possible after identification to prevent the downstream development of CVD and HF might be warranted.

## Electronic supplementary material

Below is the link to the electronic supplementary material.

Supplementary Material 1

## Data Availability

The datasets analysed in the current study are not publicly available because of agreements with the primary care organisations and Ministry of Health who provided the data but are available from the corresponding author on reasonable request.

## List of abbreviations

IGT: impaired glucose tolerance
T2D: type 2 diabetes
CVD: cardiovascular diseases
CEM: coarsened exact matching
HF: heart failure
TC: total cholesterol
LDL-C: low-density lipoprotein cholesterol
HDL-C: high-density lipoprotein cholesterol
NZ: New Zealand
SBP: Systolic blood pressure
BMI: body mass index
NZE: New Zealand European
