# Supplementary material for: Effect of onset of type 2 diabetes on risks of cardiovascular disease and heart failure among new Zealanders with impaired glucose tolerance over 25 years: tapered-matched landmark analysis
Source: Cardiovasc Diabetol. 2023 Jun 30;22:163. doi: 10.1186/s12933-023-01871-y (PMC10314599; doi:10.1186/s12933-023-01871-y)
Supplement: Supplementary file 1 — Supplementary Material 1 [file 12933_2023_1871_MOESM1_ESM.docx]

**Online Supplemental File**

**Supplemental Table 1.** ICD-9 and ICD-10 codes used to define clinical outcome

|  | ICD-9 codes | ICD-10 codes |
| --- | --- | --- |
| Cardiovascular disease | 410, 411, 412, 413, 414, 430-438 | I20-I25, I60-I69, I73 |
| Heart failure | 248, 425.4, 425.9, 402.01, 402.11, 402.91, 404.03, 404.13, 404.93, 404,01, 404.11, 404.91 | I50.0, I50.1, I50.9, I42.0, I42.9, I11.0, I25.5, I13.2, I13.0 |

**Supplemental Table 2**. Comparison of patients with and without the onset of type 2 diabetes in patients with impaired glucose tolerance

*Categorical variables were presented as count (%); continuous variables were presented as mean (standard deviation). IMD indicates the index of multiple deprivation.*

|  | **1-year landmark** | | | **2-year landmark** | | | **3-year landmark** | | | **4-year landmark** | | | **5-year landmark** | | |
| --- | --- | --- | --- | --- | --- | --- | --- | --- | --- | --- | --- | --- | --- | --- | --- |
|  | Without T2D onset | With T2D onset | *P*-value | Without T2D onset | With T2D onset | *P*-value | Without T2D onset | With T2D onset | *P*-value | Without T2D onset | With T2D onset | *P*-value | Without T2D onset | With T2D onset | *P*-value |
|  | N=24,893 | N=180 |  | N=24575 | N=381 |  | N=22703 | N=589 |  | N=19597 | N=749 |  | N=15452 | N=845 |  |
|  | **Unmatched** | | | | | | | | | | | | | | |
| Age, years | 57.3 (13.3) | 55.8 (12.9) | 0.0282 | 55.7 (12.9) | 57.7 (12.5) | 0.004 | 55.5 (12.9) | 56.3 (13.4) | 0.139 | 55.6 (12.8) | 56.2 (13.0) | 0.171 | 56.0 (12.6) | 56.4 (12.8) | 0.323 |
| Female Gender, n (%) | 13266 (53.3) | 99 (55.0) | 0.7340 | 13206 (53.7) | 201 (52.8) | 0.703 | 12201 (53.7) | 317 (53.8) | 0.970 | 10515 (53.7) | 408 (54.5) | 0.660 | 8240 (53.3) | 450 (53.3) | 0.967 |
| New Zealand European, n (%) | 12349 (49.6) | 83 (46.1) | 0.296 | 12270 (49.9) | 177 (46.5) | 0.179 | 10925 (48.1) | 254 (43.1) | 0.017 | 9611 (49.0) | 319 (42.6) | 0.001 | 8007 (51.8) | 370 (43.8) | <0.0001 |
| Enrol cohort, n (%) |  |  |  |  |  |  |  |  |  |  |  |  |  |  |  |
| 1994-1998 | 133 (0.5) | 2 (1.1) | <0.0001 | 123 (0.5) | 10 (2.6) | <0.0001 | 119 (0.5) | 12 (2.0) | <0.0001 | 117 (0.6) | 13 (1.7) | <0.0001 | 114 (0.7) | 18 (2.1) | <0.0001 |
| 1999-2003 | 558 (2.2) | 12 (6.7) |  | 536 (2.2) | 25 (6.6) |  | 526 (2.3) | 48 (8.2) |  | 518 (2.6) | 67 (9.0) |  | 510 (3.3) | 88 (10.4) |  |
| 2004-2008 | 1864 (7.4) | 35 (19.4) |  | 1815 (7.4) | 65 (17.1) |  | 1792 (7.9) | 87 (14.8) |  | 1761 (9.0) | 110 (14.7) |  | 1738 (11.3) | 160 (18.9) |  |
| 2009-2013 | 9,102 (36.6) | 69 (38.3) |  | 8959 (36.5) | 140 (36.8) |  | 8906 (39.2) | 221 (37.5) |  | 8875 (45.3) | 333 (44.5) |  | 8867 (57.4) | 441 (52.2) |  |
| 2014-2018 | 13,236 (53.2) | 62 (34.4) |  | 13142 (53.5) | 141 (37.0) |  | 11360 (50.0) | 221 (37.5) |  | 8326 (42.5) | 226 (30.2) |  | 4233 (27.3) | 138 (16.3) |  |
| IMD group (NZDep13 scale) |  |  |  |  |  |  |  |  |  |  |  |  |  |  |  |
| Least Deprivation: IMD-1 (1 or 2) | 3525 (14.6) | 18 (10.0) | 0.0280 | 3494 (14.7) | 38 (10.0) | 0.014 | 3178 (14.5) | 59 (10.0) | 0.002 | 2835 (14.9) | 76 (10.2) | <0.0001 | 2140 (14.2) | 76 (9.0) | <0.0001 |
| IMD-2 (3 or 4) | 4245 (17.6) | 20 (11.1) |  | 4196 (17.6) | 54 (14.2) |  | 3711 (16.9) | 83 (14.1) |  | 3267 (17.2) | 109 (14.6) |  | 2621 (17.4) | 128 (15.2) |  |
| IMD-3 (5 or 6) | 3040 (12.6) | 23 (12.8) |  | 2997 (12.6) | 49 (12.9) |  | 2719 (12.4) | 72 (12.2) |  | 2278 (12.0) | 93 (12.4) |  | 1912 (12.7) | 112 (13.3) |  |
| IMD-4 (7 or 8) | 3492 (14.5) | 32 (17.8) |  | 3440 (14.5) | 62 (16.3) |  | 3256 (14.8) | 90 (15.3) |  | 2908 (15.3) | 114 (15.2) |  | 2397 (16.0) | 145 (17.2) |  |
| Most Deprivation: IMD-5 (9 or 10) | 10591 (42.5) | 87 (48.3) |  | 10448 (42.5) | 178 (46.7) |  | 9839 (43.3) | 285 (48.4) |  | 8309 (42.4) | 357 (47.7) |  | 6382 (41.3) | 384 (45.4) |  |
| Smoking status, n (%) |  |  |  |  |  |  |  |  |  |  |  |  |  |  |  |
| Never smoking | 14,781 (59.4) | 98 (54.4) | 0.391 | 14623 (59.5) | 200 (52.5) | 0.022 | 13509 (59.5) | 319 (54.2) | 0.027 | 11708 (59.7) | 412 (55.0) | 0.030 | 9292 (60.1) | 470 (55.6) | 0.018 |
| Ex-smoker | 6,619 (26.6) | 53 (29.4) |  | 6514 (26.5) | 119 (31.2) |  | 5952 (26.2) | 170 (28.9) |  | 5119 (26.1) | 214 (28.6) |  | 4028 (26.1) | 235 (27.8) |  |
| Current Smoker | 3,493 (14.0) | 29 (16.1) |  | 3438 (14.0) | 62 (16.3) |  | 3242 (14.3) | 100 (17.0) |  | 2770 (14.1) | 123 (16.4) |  | 2132 (13.8) | 140 (16.6) |  |
| Body mass index, kg/m^2^ | 33.6 (7.3) | 31.2 (6.4) | <0.0001 | 31.2 (6.4) | 33.6 (6.9) | <0.0001 | 31.3 (6.4) | 33.8 (7.0) | <0.0001 | 31.2 (6.3) | 33.6 (6.8) | <0.0001 | 31.1 (6.2) | 33.6 (6.7) | <0.0001 |
| Systolic blood pressure, mmHg | 132 (17) | 131 (16) | <0.0001 | 131 (16) | 133 (17) | 0.015 | 131 (16) | 133 (17) | 0.0029 | 131 (16) | 134 (17) | <0.0001 | 132 (16) | 134 (18) | <0.0001 |
| Diastolic blood pressure, mmHg | 79 (10) | 79 (10) | 0.7180 | 79 (10) | 80 (11) | 0.016 | 79 (10) | 81 (10) | 0.003 | 79 (10) | 81 (10) | <0.0001 | 80 (10) | 81 (11) | 0.001 |
| HbA1c, mmol/mol | 42.1 (3.2) | 42.9 (3.9) | 0.003 | 42.1 (3.2) | 43.4 (3.9) | <0.0001 | 42.3 (3.2) | 44.0 (4.1) | <0.0001 | 42.4 (3.1) | 44.4 (4.0) | <0.0001 | 42.6 (3.1) | 44.5 (4.0) | <0.0001 |
| Total cholesterol, mmol/L | 5.1 (1.0) | 4.7 (1.0) | <0.0001 | 5.1 (1.0) | 4.8 (1.0) | <0.0001 | 5.1 (1.0) | 4.8 (1.0) | <0.0001 | 5.1 (1.0) | 4.9 (1.0) | <0.0001 | 5.1 (1.0) | 4.9 (1.0) | <0.0001 |
| Triglyceride, mmol/L | 1.6 (0.7) | 1.7 (0.8) | 0.3640 | 1.7 (0.8) | 1.7 (0.8) | 0.158 | 1.7 (0.8) | 1.8 (0.8) | 0.003 | 1.7 (0.8) | 1.8 (0.8) | <0.0001 | 1.6 (0.7) | 1.7 (0.8) | <0.0001 |
| Low-density lipoprotein cholesterol, mmol/L | 2.6 (0.7) | 2.8 (0.7) | <0.0001 | 2.8 (0.7) | 2.6 (0.7) | <0.0001 | 2.8 (0.7) | 2.7 (0.7) | <0.0001 | 2.8 (0.7) | 2.7 (0.8) | <0.0001 | 2.8 (0.7) | 2.7 (0.8) | <0.0001 |
| High-density lipoprotein cholesterol, mmol/L | 1.3 (0.3) | 1.3 (0.4) | 0.9810 | 1.3 (0.4) | 1.3 (0.3) | 0.150 | 1.3 (0.4) | 1.3 (0.3) | 0.002 | 1.3 (0.4) | 1.2 (0.3) | <0.0001 | 1.3 (0.4) | 1.2 (0.3) | <0.0001 |
| estimated Glomerular filtration rate<90 ml/min/1.73 m^2^ | 7875 (31.6) | 58 (32.2) | 0.0450 | 7781 (31.7) | 125 (32.8) | <0.0001 | 7414 (32.7) | 204 (34.6) | 0.003 | 6728 (34.3) | 278 (37.1) | 0.001 | 8326 (53.9) | 454 (53.7) | <0.0001 |
| Antihypertensive treatment, n (%) | 2560 (10.3) | 46 (25.6) | <0.0001 | 2486 (10.1) | 102 (26.8) | <0.0001 | 2443 (10.8) | 163 (27.7) | <0.0001 | 2375 (12.1) | 222 (29.6) | <0.0001 | 2287 (14.8) | 302 (35.7) | <0.0001 |
| Statin treatment, n (%) | 2102 (8.4) | 43 (23.9) | <0.0001 | 2042 (8.3) | 90 (23.6) | <0.0001 | 2009 (8.9) | 142 (24.1) | <0.0001 | 1959 (10.0) | 194 (25.9) | <0.0001 | 1894 (12.3) | 267 (31.6) | <0.0001 |
| Antiplatelet or anticoagulant treatment, n (%) | 75 (0.3) | 5 (2.8) | <0.0001 | 73 (0.3) | 6 (1.6) | <0.0001 | 72 (0.3) | 10 (1.7) | <0.0001 | 68 (0.4) | 12 (1.6) | <0.0001 | 67 (0.4) | 15 (1.8) | <0.0001 |
|  | **Coarsened and exact matched** | | | | | | | | | | | | | | |
| N | 2,019 | 157 |  | 3931 | 332 |  | 5163 | 508 |  | 5341 | 636 |  | 4603 | 697 |  |
| Age, years | 54.1 (13.8) | 57.6 (13.3) | 0.003 | 54.6 (13.6) | 57.8 (12.5) | <0.0001 | 53.6 (13.3) | 56.3 (13.4) | <0.0001 | 54.1 (13.2) | 56.2 (13.1) | <0.0001 | 54.0 (12.6) | 56.2 (13.0) | <0.0001 |
| Female Gender, n (%) | 1,232 (61.0) | 86 (54.8) | 0.118 | 2047 (52.1) | 172 (51.8) | 0.926 | 2742 (53.1) | 266 (52.4) | 0.748 | 2962 (55.5) | 340 (53.5) | 0.338 | 2437 (52.9) | 366 (52.5) | 0.831 |
| New Zealand European, n (%) | 597 (29.9) | 69 (44.0) | <0.0001 | 1312 (33.4) | 150 (45.2) | <0.0001 | 1613 (31.2) | 213 (41.9) | <0.0001 | 1901 (35.6) | 340 (53.5) | 0.001 | 1638 (35.6) | 303 (43.5) | <0.0001 |
| Enrol cohort, n (%) |  |  |  |  |  |  |  |  |  |  |  |  |  |  |  |
| 1994-1998 | 7 (0.4) | 2 (1.3) | <0.0001 | 12 (0.3) | 10 (3.0) | <0.0001 | 13 (0.3) | 11 (2.2) | <0.0001 | 16 (0.3) | 10 (1.6) | <0.0001 | 18 (0.4) | 15 (2.2) | <0.0001 |
| 1999-2003 | 43 (2.1) | 7 (4.5) |  | 81 (2.1) | 17 (5.1) |  | 114 (2.2) | 39 (7.7) |  | 141 (2.6) | 56 (8.8) |  | 134 (2.9) | 69 (9.9) |  |
| 2004-2008 | 106 (5.3) | 28 (17.8) |  | 230 (5.9) | 51 (15.4) |  | 303 (5.9) | 65 (12.8) |  | 401 (7.5) | 82 (12.9) |  | 417 (9.1) | 115 (16.5) |  |
| 2009-2013 | 930 (46.1) | 61 (38.9) |  | 1681 (42.8) | 123 (37.1) |  | 65 (12.8) | 193 (38.0) |  | 2906 (54.4) | 288 (45.3) |  | 3394 (73.7) | 381 (54.7) |  |
| 2014-2018 | 933 (46.2) | 59 (37.6) |  | 1927 (49.0) | 131 (39.5) |  | 193 (38.0) | 200 (39.4) |  | 1877 (35.1) | 200 (31.5) |  | 640 (13.9) | 117 (16.8) |  |
| IMD group (NZDep13 scale) |  |  |  |  |  |  |  |  |  |  |  |  |  |  |  |
| Least Deprivation: IMD-1 (1 or 2) | 145 (7.2) | 17 (10.8) | 0.021 | 334 (8.5) | 32 (9.6) | 0.001 | 432 (8.4) | 50 (9.8) | <0.0001 | 554 (10.4) | 65 (10.2) | 0.147 | 482 (10.5) | 62 (8.9) | 0.039 |
| IMD-2 (3 or 4) | 196 (9.7) | 18 (11.5) |  | 408 (10.4) | 50 (15.1) |  | 552 (10.7) | 73 (14.4) |  | 705 (13.2) | 94 (14.8) |  | 693 (15.1) | 106 (15.2) |  |
| IMD-3 (5 or 6) | 146 (7.2) | 18 (11.5) |  | 301 (7.7) | 39 (11.8) |  | 389 (7.5) | 58 (11.4) |  | 482 (9.0) | 74 (11.6) |  | 467 (10.2) | 89 (12.8) |  |
| IMD-4 (7 or 8) | 311 (15.4) | 29 (18.5) |  | 605 (15.4) | 54 (16.3) |  | 822 (15.9) | 77 (15.2) |  | 823 (15.4) | 96 (15.1) |  | 677 (14.7) | 121 (17.4) |  |
| Most Deprivation: IMD-5 (9 or 10) | 1,221 (60.5) | 75 (47.8) |  | 2283 (58.1) | 157 (47.3) |  | 2968 (57.5) | 250 (49.2) |  | 2777 (52.0) | 307 (48.3) |  | 2284 (49.6) | 319 (45.8) |  |
| Smoking status, n (%) |  |  |  |  |  |  |  |  |  |  |  |  |  |  |  |
| Never smoking | 1,095 (54.2) | 82 (52.2) | 0.802 | 2045 (52.1) | 175 (52.7) | 0.814 | 2883 (55.8) | 278 (54.7) | 0.848 | 3191 (59.8) | 355 (55.8) | 0.152 | 2719 (59.1) | 386 (55.4) | 0.183 |
| Ex-smoker | 566 (28.0) | 48 (30.6) |  | 1167 (29.7) | 101 (30.4) |  | 1384 (26.8) | 142 (28.0) |  | 1316 (24.6) | 175 (27.5) |  | 1148 (24.9) | 189 (27.1) |  |
| Current Smoker | 358 (17.7) | 27 (17.2) |  | 718 (18.3) | 56 (16.9) |  | 896 (17.4) | 88 (17.3) |  | 834 (15.6) | 106 (16.7) |  | 736 (16.0) | 122 (17.5) |  |
| Body mass index, kg/m^2^ | 33.8 (6.6) | 33.6 (7.3) |  | 33.4 (6.5) | 33.5 (7.0) | 0.944 | 33.2 (6.5) | 33.7 (6.9) | 0.150 | 33.0 (6.4) | 33.5 (6.7) | 0.069 | 32.7 (6.5) | 33.8 (6.7) | 0.002 |
| Systolic blood pressure, mmHg | 132 (17) | 132 (16) | 0.773 | 134 (17) | 133 (17) | 0.579 | 132 (17) | 133 (17) | 0.378 | 133 (16) | 133 (17) | 0.0388 | 132 (16) | 134 (17) | 0.0153 |
| Diastolic blood pressure, mmHg | 80 (10) | 79 (10) | 0.125 | 81 (10) | 80 (11) | 0.352 | 81 (10) | 81 (10) | 0.570 | 81 (10) | 81 (10) | 0.320 | 81 (10) | 81 (11) | <0.0001 |
| HbA1c, mmol/mol | 43.2 (2.8) | 43.1 (3.8) | 0.762 | 43.2 (2.8) | 43.7 (3.8) | 0.010 | 43.3 (2.8) | 44.2 (4.0) | <0.0001 | 43.4 (2.8) | 44.5 (3.9) | <0.0001 | 43.4 (2.8 | 44.8 (3.8) | <0.0001 |
| Total cholesterol, mmol/L | 5.1 (0.9) | 4.8 (1.0) | <0.0001 | 5.2 (0.9) | 4.8 (1.0) | <0.0001 | 5.1 (0.9) | 4.9 (1.0) | <0.0001 | 5.2 (0.9) | 4.9 (1.0) | <0.0001 | 5.2 (0.9) | 4.9 (1.0) | <0.0001 |
| Triglyceride, mmol/L | 1.7 (0.8) | 1.7 (0.7) | 0.349 | 1.8 (0.8) | 1.8 (0.8) | 0.743 | 1.7 (0.8) | 1.8 (0.8) | 0.196 | 1.7 (0.8) | 1.8 (0.8) | 0.046 | 1.7 (0.8) | 1.8 (0.8) | <0.0001 |
| Low-density lipoprotein cholesterol, mmol/L | 2.8 (0.7) | 2.6 (0.7) | <0.0001 | 2.9 (0.7) | 2.6 (0.7) | <0.0001 | 2.9 (0.7) | 2.7 (0.8) | 0.027 | 2.9 (0.7) | 2.7 (0.8) | <0.0001 | 2.9 (0.7) | 2.7 (0.7) | <0.0001 |
| High-density lipoprotein cholesterol, mmol/L | 1.3 (0.4) | 1.3 (0.3) | 0.081 | 1.3 (0.4) | 1.3 (0.3) | 0.596 | 1.3 (0.4) | 1.3 (0.3) | 0.768 | 1.3 (0.4) | 1.3 (0.3) | 0.113 | 1.3 (0.4) | 1.2 (0.3) | <0.0001 |
| estimated Glomerular filtration rate<90 ml/min/1.73 m^2^ | 907 (44.9) | 50 (31.9) | 0.005 | 1417 (36.1) | 106 (31.9) | 0.073 | 1907 (36.9) | 170 (33.5) | 0.247 | 2123 (39.8) | 232 (36.5) | 0.052 | 3171 (69.9) | 395 (56.7) | <0.0001 |
| Antihypertensive treatment, n (%) | 145 (7.2) | 35 (22.3) | <0.0001 | 343 (8.7) | 75 (22.6) | <0.0001 | 492 (9.5) | 123 (24.2) | <0.0001 | 615 (11.5) | 164 (25.8) | <0.0001 | 657 (14.3) | 223 (32.0) | <0.0001 |
| Statin treatment, n (%) | 121 (6.0) | 34 (21.7) | <0.0001 | 272 (6.9) | 71 (21.4) | <0.0001 | 396 (7.7) | 109 (21.5) | <0.0001 | 491 (9.2) | 147 (23.1) | <0.0001 | 545 (11.8) | 200 (28.7) | <0.0001 |
| Antiplatelet or anticoagulant treatment, n (%) | 11 (0.5) | 5 (3.2) | <0.0001 | 14 (0.4) | 6 (1.8) | <0.0001 | 18 (0.4) | 7 (1.4) | <0.0001 | 21 (0.4) | 8 (1.3) | <0.0001 | 18 (0.4) | 10 (1.4) | <0.0001 |

**Supplemental Figure S1**. Workflow charts for matching process (1-year landmark analysis)


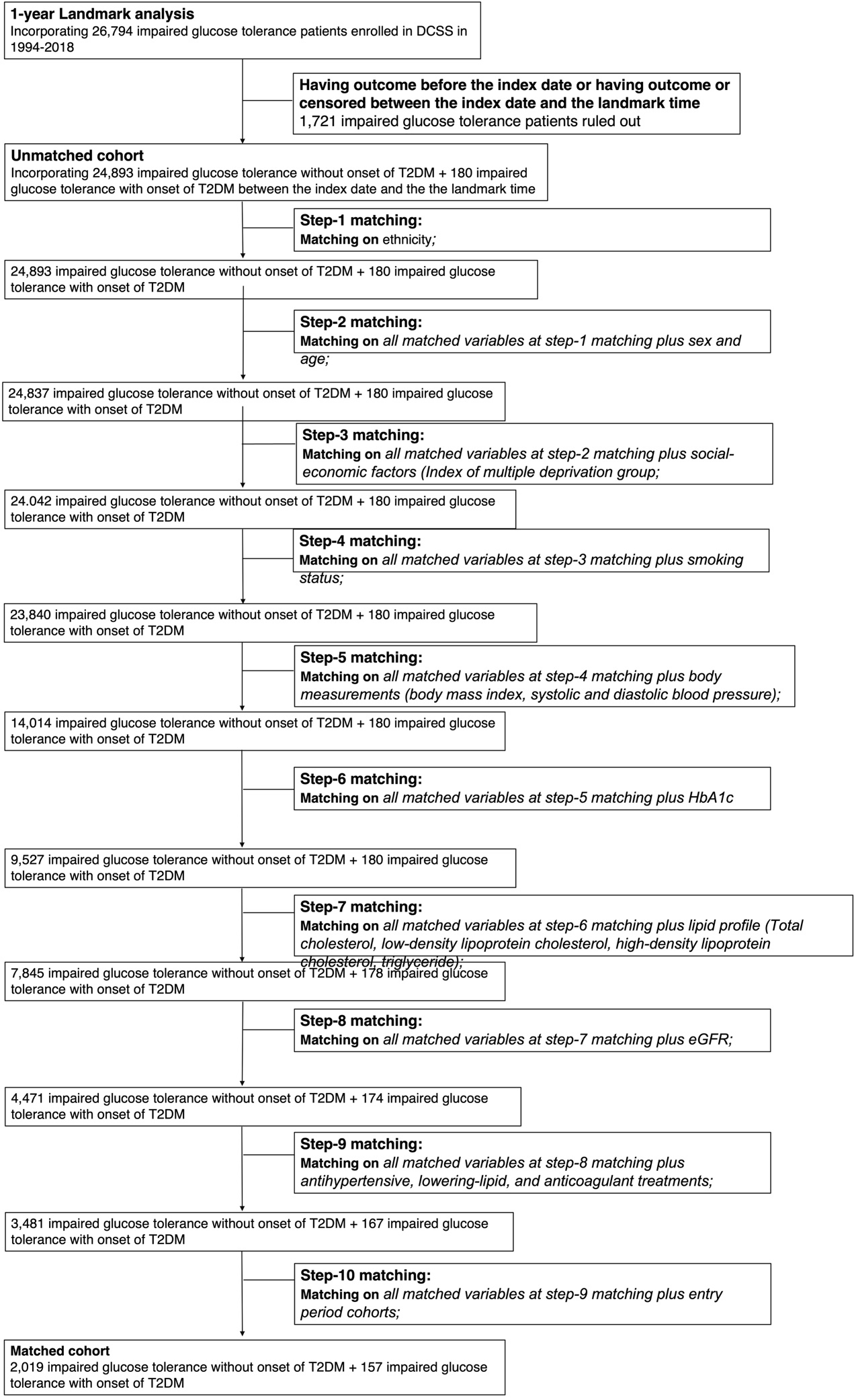


**Supplemental Figure S2**. Workflow charts for matching process (2-year landmark analysis)


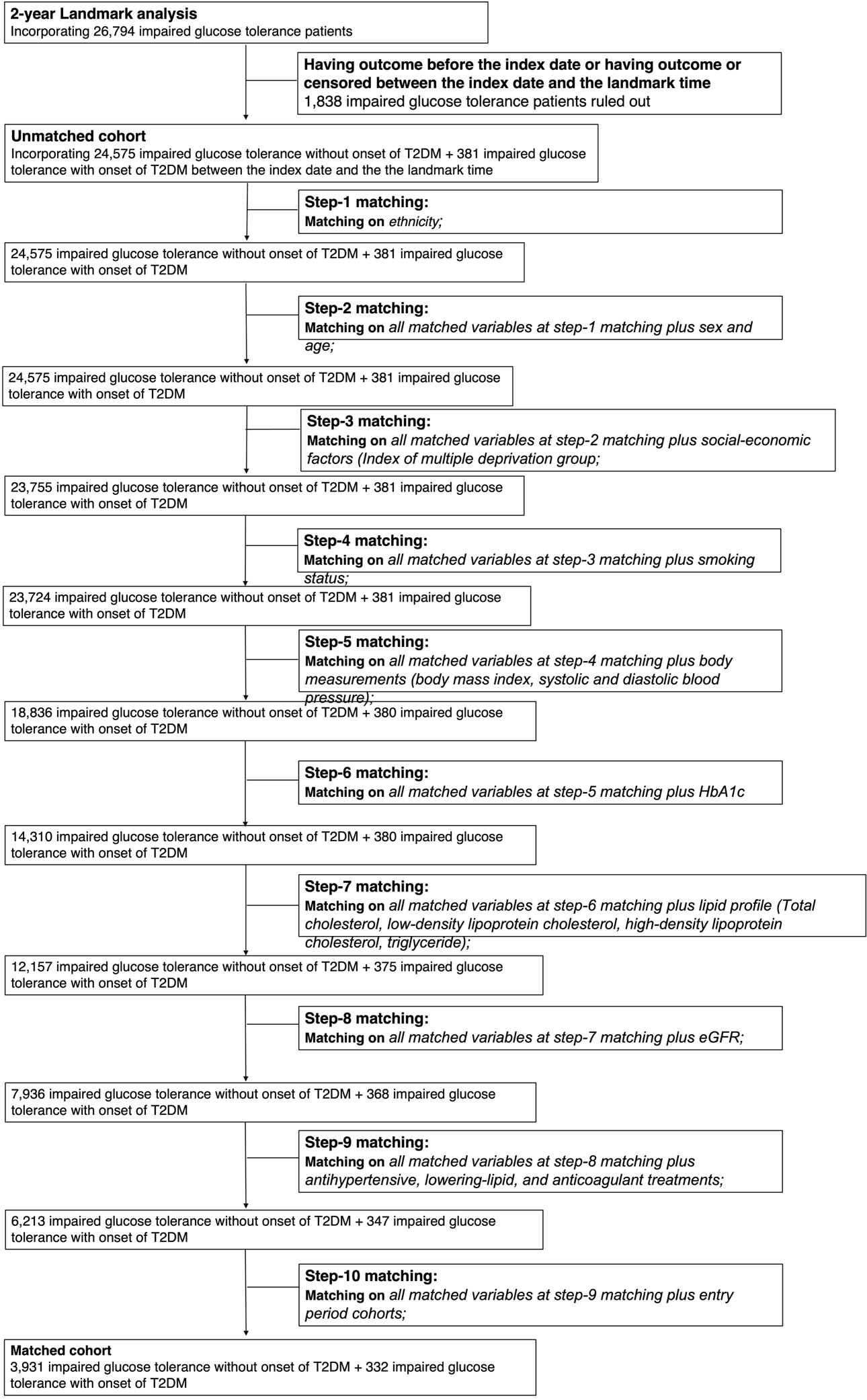


**Supplemental Figure S3**. Workflow charts for matching process (3-year landmark analysis)


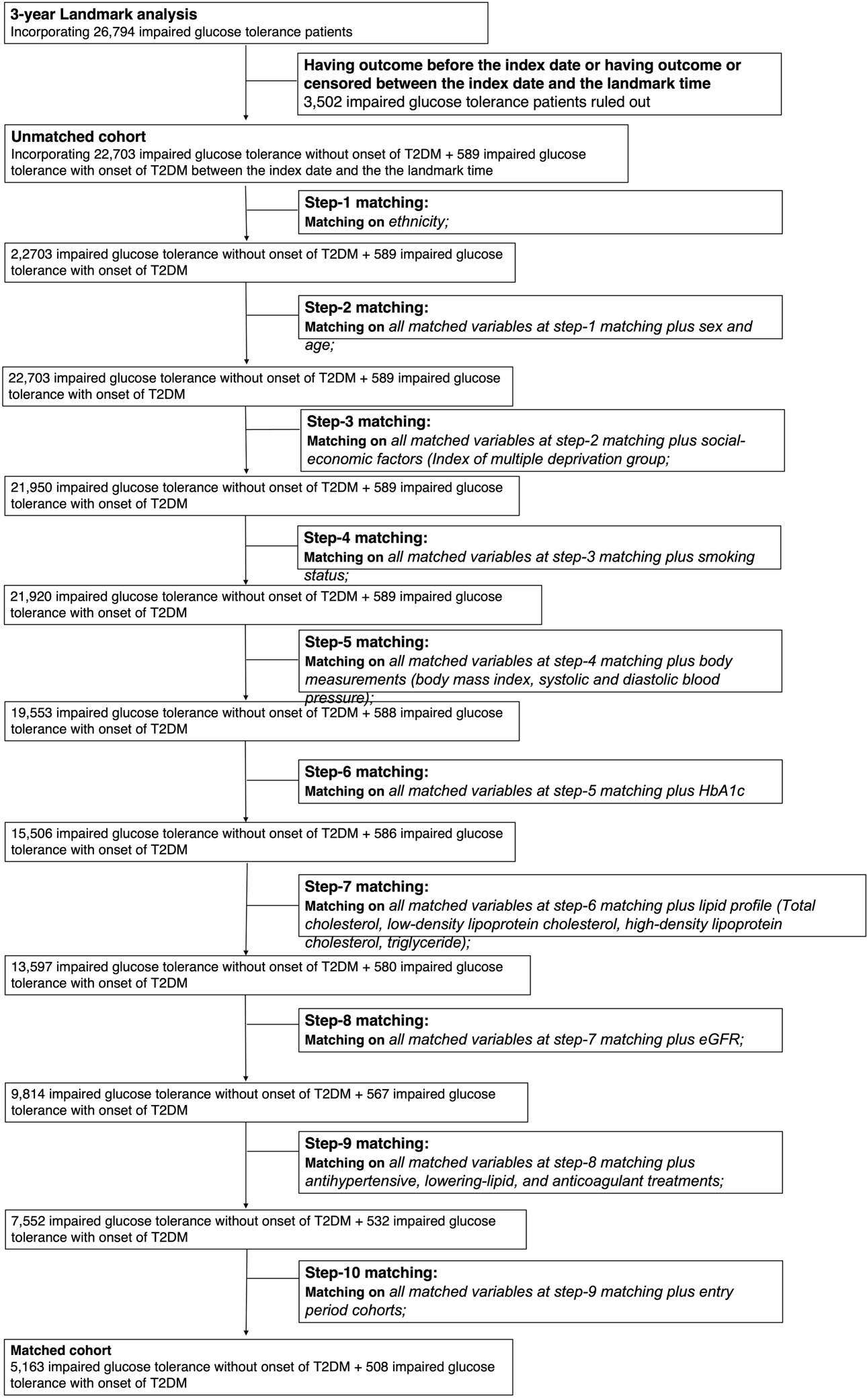


**Supplemental Figure S4**. Workflow charts for matching process (4-year landmark analysis)


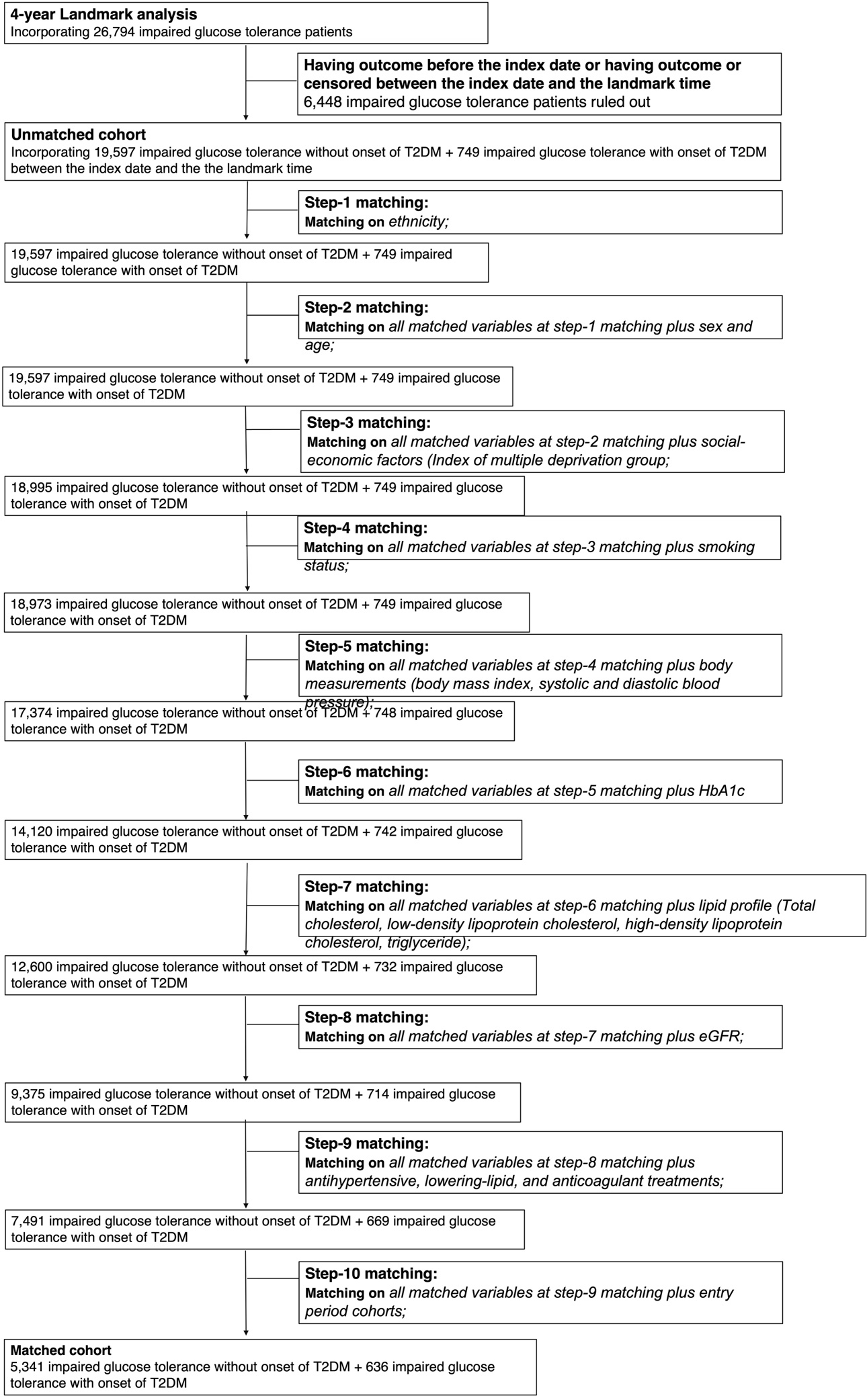


**Supplemental Figure S5**. Workflow charts for matching process (5-year landmark analysis)


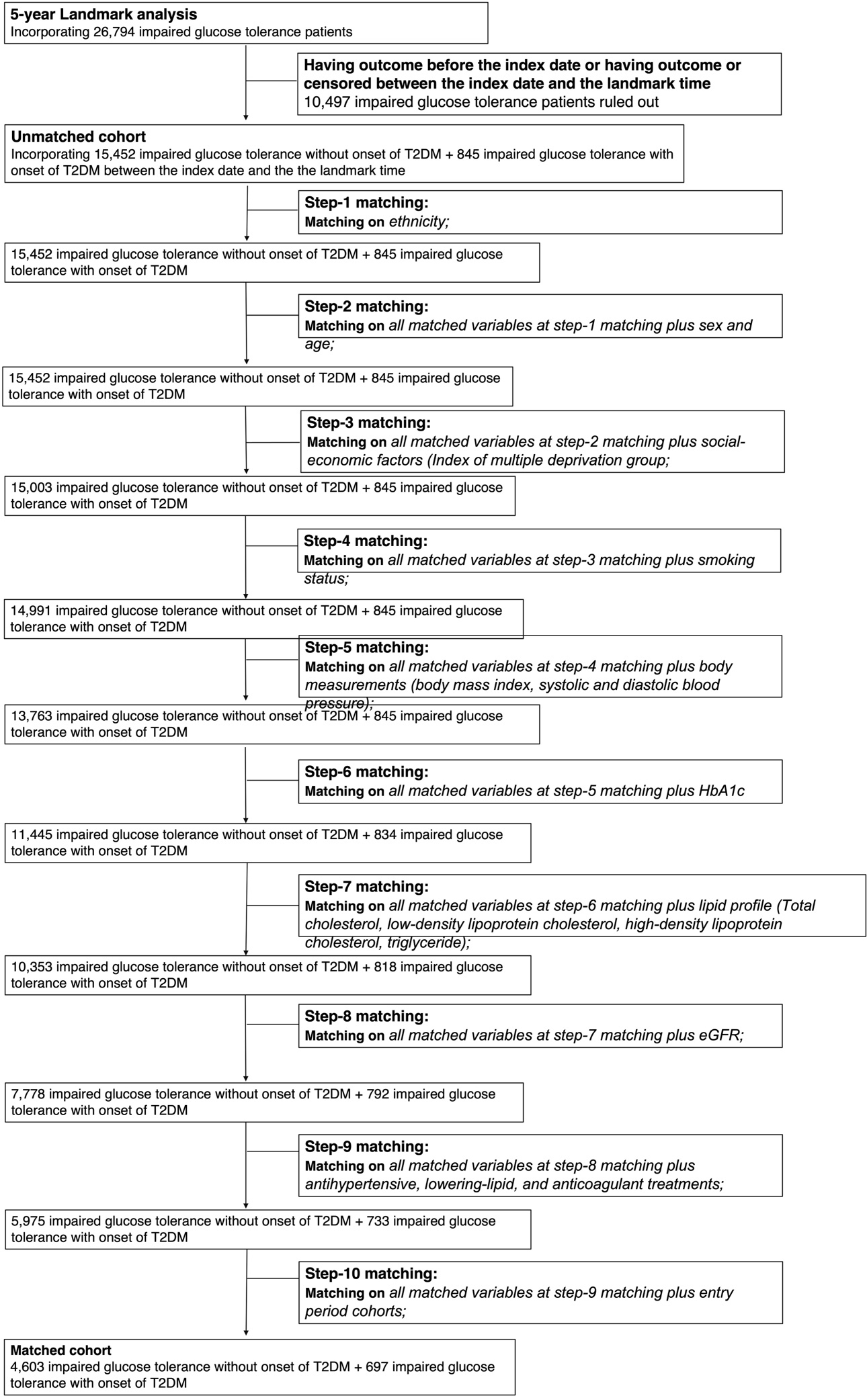


**Supplemental Figure S6**. Adjusted risk ratios for 5-year risk of cardiovascular disease between patients with impaired glucose tolerance with and without the onset of type 2 diabetes

*Model (i) weighted for ethnicity; model (ii) weighted for all adjusted variables in model (i) plus age and sex; model (iii) weighted for all adjusted variables in model (ii) plus IMD group; model (iv) weighted for all adjusted variables in model (iii) plus smoking status; model (v) weighted for all adjusted variables in model (iv) plus body measurements (body mass index, systolic and diastolic blood pressure); model (vi) weighted for all adjusted variables in model (v) plus baseline HbA1c; model (vii) weighted for all adjusted variables in model (vi) plus baseline lipid profile (total cholesterol, low-density lipoprotein cholesterol, high-density lipoprotein cholesterol, and triglyceride); model (viii) weighted for all adjusted variables in model (vii) plus eGFR; model (ix) weighted for all adjusted variables in model (viii) plus antihypertensive, lowering lipid and anticoagulant treatment; model (x) weighted for all adjusted variables in model (ix) plus entry cohorts; The log-scale for Y-axis (risk ratio) was applied.*

**Supplemental Figure S7**. Adjusted risk ratios for 10-year risk of cardiovascular diseases between people with impaired glucose tolerance with and without the onset of type 2 diabetes

*Model (i) weighted for ethnicity; model (ii) weighted for all adjusted variables in model (i) plus age and sex; model (iii) weighted for all adjusted variables in model (ii) plus IMD group; model (iv) weighted for all adjusted variables in model (iii) plus smoking status; model (v) weighted for all adjusted variables in model (iv) plus body measurements (body mass index, systolic and diastolic blood pressure); model (vi) weighted for all adjusted variables in model (v) plus baseline HbA1c; model (vii) weighted for all adjusted variables in model (vi) plus baseline lipid profile (total cholesterol, low-density lipoprotein cholesterol, high-density lipoprotein cholesterol, and triglyceride); model (viii) weighted for all adjusted variables in model (vii) plus eGFR; model (ix) weighted for all adjusted variables in model (viii) plus antihypertensive, lowering lipid and anticoagulant treatment; model (x) weighted for all adjusted variables in model (ix) plus entry cohorts; The log-scale for Y-axis (risk ratio) was applied.*

**Supplemental Figure S8**. Adjusted risk ratios for 5-year risk of heart failure between patients with impaired glucose tolerance with and without the onset of type 2 diabetes

*Model (i) weighted for ethnicity; model (ii) weighted for all adjusted variables in model (i) plus age and sex; model (iii) weighted for all adjusted variables in model (ii) plus IMD group; model (iv) weighted for all adjusted variables in model (iii) plus smoking status; model (v) weighted for all adjusted variables in model (iv) plus body measurements (body mass index, systolic and diastolic blood pressure); model (vi) weighted for all adjusted variables in model (v) plus baseline HbA1c; model (vii) weighted for all adjusted variables in model (vi) plus baseline lipid profile (total cholesterol, low-density lipoprotein cholesterol, high-density lipoprotein cholesterol, and triglyceride); model (viii) weighted for all adjusted variables in model (vii) plus eGFR; model (ix) weighted for all adjusted variables in model (viii) plus antihypertensive, lowering lipid and anticoagulant treatment; model (x) weighted for all adjusted variables in model (ix) plus entry cohorts; The log-scale for Y-axis (risk ratio) was applied.*

**Supplemental Figure S9**. Adjusted risk ratios for 10-year risk of heart failure between patients with impaired glucose tolerance with and without the onset of type 2 diabetes

*Model (i) weighted for ethnicity; model (ii) weighted for all adjusted variables in model (i) plus age and sex; model (iii) weighted for all adjusted variables in model (ii) plus IMD group; model (iv) weighted for all adjusted variables in model (iii) plus smoking status; model (v) weighted for all adjusted variables in model (iv) plus body measurements (body mass index, systolic and diastolic blood pressure); model (vi) weighted for all adjusted variables in model (v) plus baseline HbA1c; model (vii) weighted for all adjusted variables in model (vi) plus baseline lipid profile (total cholesterol, low-density lipoprotein cholesterol, high-density lipoprotein cholesterol, and triglyceride); model (viii) weighted for all adjusted variables in model (vii) plus eGFR; model (ix) weighted for all adjusted variables in model (viii) plus antihypertensive, lowering lipid and anticoagulant treatment; model (x) weighted for all adjusted variables in model (ix) plus entry cohorts; The log-scale for Y-axis (risk ratio) was applied.*
